# Local and Remote Effects of Mesenchymal Stem Cell Administration on Skin Wound Regeneration

**DOI:** 10.3390/pathophysiology28030024

**Published:** 2021-08-12

**Authors:** Ekaterina Silina, Victor Stupin, Konstantin Koreyba, Sergey Bolevich, Yulia Suzdaltseva, Natalia Manturova

**Affiliations:** 1 Department of Human Pathology, I.M. Sechenov First Moscow State Medical University (Sechenov University), 119991 Moscow, Russia; bolevich2011@yandex.ru; 2 Department of Hospital Surgery №1, N.I. Pirogov Russian National Research Medical University (RNRMU), 117997 Moscow, Russia; stvictor@bk.ru (V.S.); korejba_k@mail.ru (K.K.); 3 Department of Human and Animal Epigenetics, Vavilov Institute of General Genetics of the Russian Academy of Sciences, 119333 Moscow, Russia; yu_suzdaltseva@mail.ru; 4 Department of Plastic and Reconstructive Surgery, Cosmetology and Cell Technologies, N.I. Pirogov Russian National Research Medical University, 117997 Moscow, Russia; manturovanatali@yandex.ru

**Keywords:** regeneration, mesenchymal stem cells, human umbilical cord, skin wound, wound healing, inflammation, leukocytes, fibroblasts, local and remote stem cell effects

## Abstract

Wound healing is an important medical problem. We evaluated the efficacy of locally administered mesenchymal stem cells (MSCs) isolated from human umbilical cords on the dynamics of skin wound healing. The study was conducted on the backs of Wistar rats, where two square wounds were created by removing all layers of the skin. Four groups were studied in two series of experiments: (1) a Control_NaCl group (the wounds were injected with 0.9% NaCl solution) and a Control_0 group (intact wounds on the opposite side of the same rat’s back); (2) an MSC group (injected MSCs, local effect) and a Control_sc group (intact wounds on the opposite side of the back, remote MSC effect). The area and temperature of the wounds and the microcirculation of the wound edges were measured. Histological and morphometric studies were performed on days 3 and 7 after the wounds were created. The results showed that the injection trauma (Control_NaCl) slowed the regeneration process. In both MSC groups (unlike in either control group), we observed no increase in the area of the wounds; in addition, we observed inhibition of the inflammatory process and improved wound regeneration on days 1–3 in the remote group and days 1–5 in the local (injected) group. The MSC and Control_sc groups demonstrated improved microcirculation and suppression of leukocyte infiltration on day 3. On day 7, all the studied parameters of the wounds of the Control_0 group were the same as those of the wounds that received cell therapy, although in contrast to the results of the Control_ NaCl group, fibroblast proliferation was greater in the MSC and Control_sc groups. The dynamics of the size of the wounds were comparable for both local and remote application of MSCs. Thus, even a one-time application of MSCs was effective during the first 3–5 days after injury due to anti-inflammatory processes, which improved the regeneration process. Remote application of MSC, as opposed to direct injection, is advisable, especially in the case of multiple wounds, since the results were indistinguishable between the groups and injection trauma was shown to slow healing.

## 1. Introduction

The versatile nature of stem cells makes them desirable for use in human and veterinary medicine. The number of mesenchymal stem cell (MSC) studies has been increasing. It is now widely accepted that MSCs exert pleiotropic therapeutic effects predominantly via the secretion of soluble factors including proteins, nucleic acids, lipids, and extracellular vesicles, which perform anti-inflammatory, immunomodulatory, chemoattractive, proangiogenic, anti-apoptotic, anti-oxidative, and anti-fibrotic functions in damaged tissues, promoting the healing process [1,2,3,4]. Previous studies suggested that MSCs can enhance angiogenesis through the secretion of proangiogenic factors, which promote the migration and proliferation of endothelial cells and support the stabilization and maturation of newly formed vessels [2,5,6,7]. The MSCs-mediated secretion of matrix proteins such as fibronectin and collagen also generates a microenvironment favorable for epithelial growth [8,9]. MSCs were also shown to exhibit immunomodulatory effects due to the induction of functional changes in immune cells and the ability to regulate the balance between pro-inflammatory and anti-inflammatory factors produced by them [10,11,12,13]. The differentiation of MSCs into myofibroblasts may contribute to wound contraction [14,15]. The results of these studies should lead to the creation of new and effective methods to treat a variety of diseases. The conclusions of some authors performing meta-analyses and systematic reviews of MSCs’ effectiveness at the preclinical stage seem to be encouraging [16,17,18]; conversely, the results from several clinical systematic reviews and meta-analyses are not [19,20,21,22]. Thus, the efficacy observed during research has not yet provided the basis for a practical medicinal breakthrough. It is possible that this is due not to the complexity of cell-life-regulation mechanisms alone, but rather to the difficulties in modeling somatic diseases. Perhaps the only accurate biological model for this research is an acute wound model.

Although many animal studies used MSCs as a treatment to heal skin wounds [23,24,25,26,27,28,29,30], currently, no systematic reviews or meta-analyses have demonstrated high-quality efficacy of MSC treatment. In addition, no widely accepted method exists for introducing MSCs into an animal organism. In these studies, most of the wounds were simulated in young mice that were then locally treated with stem cells.

We hypothesized that local administration of MSCs can have both local and systemic remote (general) stimulating effects on the body, promoting accelerated wound healing due to the appearance in the general organ bloodstream of the biologically active substances synthesized by MSCs.

Therefore, the aim of our study was to assess the local and remote effects of treatment with MSCs isolated from human umbilical cords on the speed and dynamics of skin wound healing in elderly rats. In this study, we inflicted two wounds on both sides of the back, and MSCs were injected locally into the area of only one wound once. The dynamics of the healing of this wound were used to assess the local effect of MSCs injection. A wound on the opposite side of the same animal served to assess the remote (general, systemic) effect of MSC administration.

It is known that aging reduces the capacity for tissue regeneration [31,32,33]. This is true for both the skeletal system and soft tissues. Inflammatory and microvascular changes in old tissues lead to longer wound healing, which is why we used elderly animals in this study. Additionally, our findings show that changes occur during the first stages of regeneration, the success and duration of which determine whether the wound will become chronic or heal and whether the scar will be of high quality and aesthetically acceptable [33,34,35,36]. Application of MSCs showed promising results in gerontology because of the anti-inflammatory effect mediated through cytokines, growth factors, and neoangiogenesis stimulation, all of which are particularly important during the first (inflammatory) stage of wound healing [27,28,37,38].

In our work, we used a rodent model of acute full-thickness wounds, considering the experience of previous similar studies using human MSCs [28,39,40,41]. We also relied on the recently confirmed hypothesis that not only live MSCs but also apoptotic cells and even cell fragments are capable of exerting a positive therapeutic effect [13]. In addition, we chose to use human umbilical cord MSCs in animal studies because, in the future, these cells, cultivated according to the patented technology described below, will be used in clinical studies involving humans as allogeneic transplants.

## 2. Materials and Methods

Fifty-six 9-month-old male Wistar rats (423 ± 53 g) were used in this study. In choosing an animal model for experiments, we were guided by the experience of previous studies as well as considerations of economic feasibility. We used old rats in the experiment because, with ageing, the potential for regeneration weakens; problems with long-term non-healing wounds mainly occur in the second half of life. There were no fundamental considerations regarding the choice of the sex of animals, since the regeneration processes depend to a much greater extent on age rather than sex characteristics. We chose male rats because there were enough of them available in the age range we needed and they were less expensive than female rats.

This rat study was approved by the Regional Ethics Committee of the Kursk State Medical University under the Ministry of Health of the Russian Federation (Protocol No. 5 dated 2 November 2017).

The wounds were created under non-sterile conditions using general anesthesia (chloral hydrate, 300 mg/kg, injected intraperitoneally). On the shaved skin of the rats’ backs, at the same distance on both sides of the spine, wounds of specific sizes were formed (square wounds, wound-to-fascia depth). The initial area of the wounds under investigation 10–20 min after the wounding was 130 mm^2^ on average (133.4 ± 2.1 mm^2^).

Inflammatory phase assessments were performed during the first week after modeling. The control points were day 0 (modeling wounds, recording measurements, and injecting MSC or physiological saline, depending on the randomization group), and days 1, 3, 5, and 7 (evaluation of the wound healing dynamics). Histological examination of six to seven wounds in each group was performed on post-wound days 3 and 7. At the end of the experiments, the animals were euthanized under general anesthesia (chloral hydrate, 300 mg/kg, injected intraperitoneally).

Before the experiment, the animals were kept in quarantine for 2 weeks. After that, the animals were randomized by weight into the groups for the two series in the experiment.

### 2.1. Groups

Four groups were studied in 2 series of experiments. In both series, animals were the same age (9 months), sex (male), and weight (423 ± 53 g).

**Figure 1:**
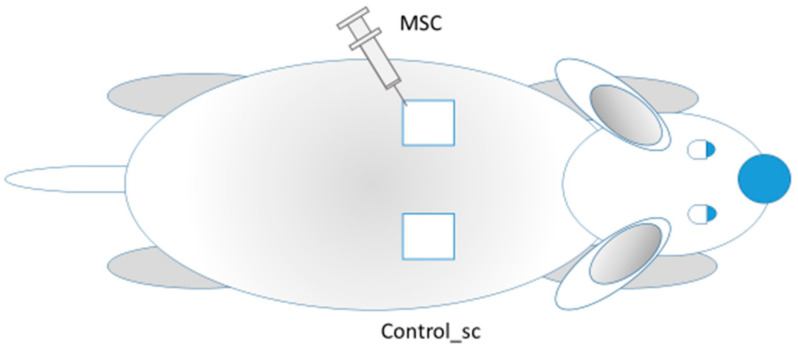
MSCs injections into the left wound (MSC group); the other wound remained intact (Control_sc group).

In the first series, 56 wounds were modeled in 28 animals to test the efficacy of local MSCs administration and its remote effect on skin wound healing. Stem cells (MSC group) were injected on the day of wounding (day 0) at the edges of the wound. The concentration of the progenitor MSC culture of a human umbilical cord solution was 0.5 × 10^6^ per mL. Through a single puncture (in the lower lateral edge of the wound), aliquots of 0.1 mL were injected in the lateral edge and 0.1 mL in the caudal edge for a total of 100,000 MSCs per wound. The remote effect of MSCs (generalization of MSCs action) was assessed by observing the intact contralateral wounds located on the opposite side of the body of the same rat (Control_sc group) (Figure 1). Thus, on one rat, we studied the healing of the skin wound after injection of MSCs (local action of MSCs) into the wound on one side; on the other side of the back of the same rat, nothing was injected directly into the wound (the general effect MSCs).

The second series of the experiment also included 28 animals (56 control wounds). The wounds of the Control_0 group were not treated, remained intact throughout the experiment, and healed under scabs. On the wounding day, an isotonic saline solution of sodium chloride (0.9% NaCl) was injected into the edges of the wounds of the Control_NaCl group, thus imitating anesthesia during the surgical treatment. Overall, four groups of wounds were studied: Control_0, Control_NaCl, Control_sc, and MSC groups.

### 2.2. Isolation and Culture of Human Umbilical Cord Mesenchymal Stem Cells

Human umbilical cords were collected after normal delivery of 38–40 weeks of gestation infants from healthy donors who signed voluntary informed consent forms. The umbilical cord fragments were washed with Hanks’ solution (HyClone, Cytiva, Marlborough, MA, USA), disintegrated, and treated with 0.1% I type collagenase solution (200 U/mL, Worthington Biochemical, Lakewood, NJ, USA) in DMEM/F12 medium (HyClone, Cytiva, Marlborough, MA, USA) for 60 min at 37 °C. The samples were harvested by centrifugation for 5 min at 260× *g*. The pellet was re-suspended in a medium supporting the growth of undifferentiated MSCs (Advance Stem Cell Basal Medium, HyClone, Cytiva, Marlborough, MA, USA) containing a 10% mixture of growth factors (Advance Stem Cell Growth Supplement, HyClone, Cytiva, Marlborough, MA, USA), 100 U/mL penicillin, and 100 U/mL streptomycin (HyClone, Cytiva, Marlborough, MA, USA), to a concentration of 10^4^ tissue fragments/mL. The suspension was transferred into culture dishes and incubated at 37 °C in a humidified atmosphere containing 5% CO_2_ until ~70%–80% confluence was reached. Umbilical cord MSC (UCMSC) cultures were expanded to passage 3 before being used for further experiments. Less than 30 min before animal injection, UCMSCs were dissociated with trypsin/EDTA solution (Gibco, Thermo Fisher Scientific, Waltham, MA, USA) and washed with Dulbecco’s phosphate-buffered saline (DPBS, HyClone, Cytiva, Marlborough, MA, USA). The cell pellet was re-suspended in sterile saline to a concentration of 1 × 10^6^ cells/mL. Viable cell counts were obtained using a TC20 cell counter (BioRad, Hercules, CA, USA) [42].

**Figure 2:**
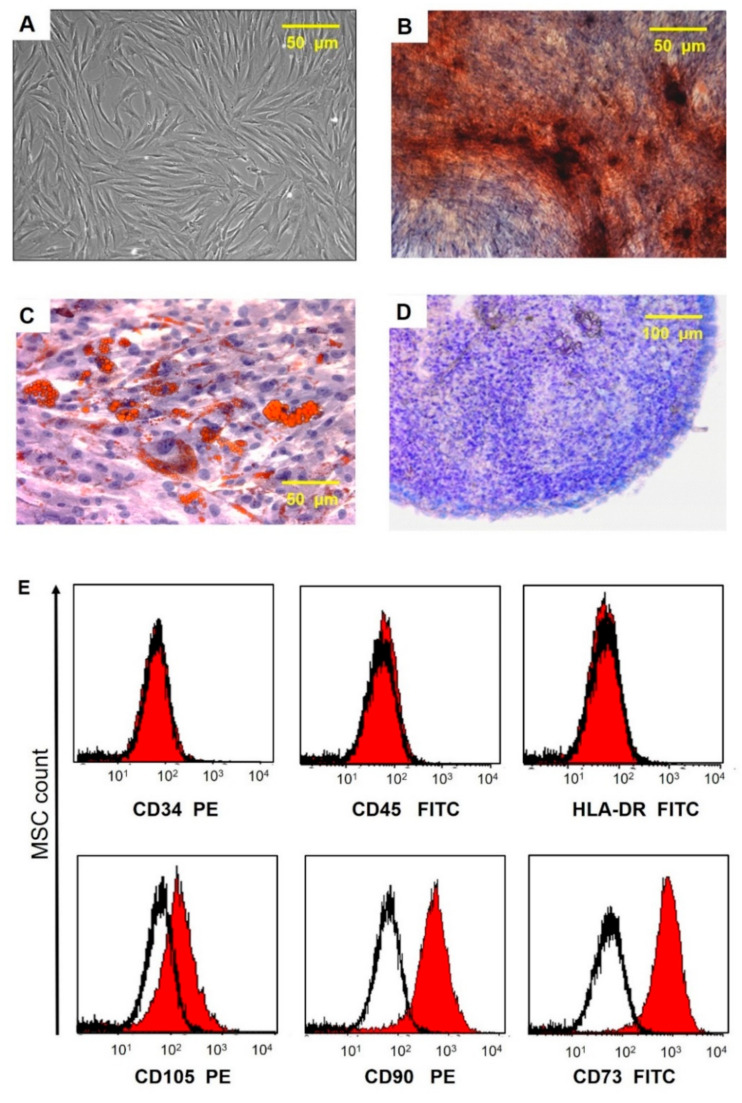
Morphological characteristics, immunocytophenotypic profile, and differentiation potential of umbilical cord MSCs. (**A**) Phase contrast image of adherent MSC with fibroblast-like morphology is shown at passage 3. (**B**) Calcium deposits in MSC (staining with alizarin red). (**C**) Lipid vacuole formation in MSCs (staining with oil red and hematoxylin); (**D**) Mucopolysaccharide extracellular matrix formation (cryosections of micromasses stained with toluidine blue). (**E**) Flow fluorometry histograms demonstrating the expression of surface antigens in MSC labeled with FITC- and PE-conjugated antibodies to surface markers CD90, CD73, CD105, CD34, CD45, and HLA-DR (red area) are plotted against isotype control (black line).

After passage 3, the cell-surface antigen profiles of adherent cells in culture were analyzed by flow cytometry. Immunocytochemical staining of cells with antibodies against the human antigens CD105, CD73, CD90, CD34, CD45, and HLA-DR conjugated with fluorochrome phycoerythrin (PE)and fluorescein isothiocyanate (FITC) (BD Bioscience, USA) was performed according to the manufacturer’s instructions. Samples were analyzed using a FACS CANTO II flow cytometer (BD Biosciences, San Jose, CA, USA). The data were processed using the FLOWJO software package (Figure 2). The ability of UCMSCs to differentiate into adipogenic, osteogenic, and chondrogenic lineages was evaluated as we described previously [43].

### 2.3. Research Methods

During dynamics analysis, the area of the wound, the temperature of the center of the wound, the microhemocirculation of the wound edges, and the weight of rats were measured. The wound area (in mm^2^), precisely limited by its edges, was recorded using the JMicroVision 1.2.7 program (Switzerland). For that, the wounds (Canon EOS550D, Japan, Jpeg format) were photo-documented 30 cm from the object, with the same foreshortening. The lobar wound area change index was calculated according to the formula:

(S_x_ − S_0_) ÷ S_0_ × 100%, (1)

where S_0_ is the wound surface area on the wounding day; S_x_ is the wound surface area on control days 1, 3, 5, or 7; and the final result is the percentage of the initial wound size.

A Fluke VT02 infrared thermometer (Fluke, Everett, WA, USA) was used to assess the temperature of the tissue at the center of each wound. Animals were weighed using a Supra BSS-4095 weighing pan (Supra, Hong Kong).

Microhemocirculation was studied in anesthetized rats on days 0, 3, and 7 using the MP150 hardware and software package for electrophysiological studies (BIOPAC Systems Inc., Goleta, CA, USA), which includes an LDF100C module for laser Doppler flowmetry, a Doppler flowmeter TSD145 laser needle, and AcqKnowledge version 4.4.1 software. The system detects the shift in the reflected signal frequency and, under the programmed signal processing algorithm, translates frequency shifts into perfusion units (blood perfusion units (BPU)). A needle probe was set perpendicular to the skin 2 mm from each wound edge and centered over the wound.

For histological analysis, 5 µm thick wound sections were investigated using light microscopy. For each wound, at least three consecutive sections were cut and then stained with hematoxylin and eosin (for descriptive light microscopy) or hematoxylin alone (for processing using the Image J program (National Institutes of Health, Bethesda, MD, USA) with quantitative analysis of resident and non-resident cell numbers). During analysis of the wound sections, the wounds were divided into three sectors (center and two opposite edges). Each sector occupied 33.3% of the total transverse size of the wound. The wound edge was marked by preserved tissue structures, including mature collagen fibers and intact skin. On histological sections, we assessed the number of cells and their character in all granulation layers at the center and on edges of the wounds. The Image-J 1.8.0_112 morphometry package (National Institutes of Health), used by morphologists for image analysis and processing, was used during the analysis [44,45]. The program is written in Java. It determines the density and number of cells per unit area, including the discretization of cells by their generally accepted morphometric visual characteristics based on the size and shape of the cells and their nuclei. Eventually, two main types of cells were obtained: fibroblasts (resident cells formed in the tissue) and leukocytes (non-resident cells that migrated to the tissue). Their absolute value (number of cells per mm^2^), as well as the ratio, which indicates the phase of healing, were analyzed. Descriptive microscopy at magnifications of 40×, 100×, and 400× was performed using a Levenhuk D740 microscope (Levenhuk, Tampa, FL, USA).

Statistical analysis was performed using SPSS 23.0 software (IBM SPSS Statistics for Windows, IBM Corp., Armonk, NY, USA), and standard parametric and nonparametric criteria were applied. Descriptive statistics of continuous quantitative data are presented as the mean, standard error of the mean, median, and values of the lower (25%) and upper (75%) quartiles. Four independent nonparametric samples were compared by performing the Kruskal–Wallis test. Two independent nonparametric samples were compared by performing the Mann–Whitney U-test, and two dependent nonparametric samples were compared by performing the Wilcoxon test. The differences were considered reliable at *p* < 0.05.

## 3. Results

### 3.1. Dynamics of Wound Tissue Area Healing

The wound area of the Control_0 group significantly increased during the first 24 h from modeling. The average increase was 26.5 mm^2^, or 17.7% (*p* < 0.05). By day 3, the wound areas of the intact control wounds decreased to return to their initial sizes. By day 5, the average wound area of the intact control wounds decreased by 9.4% (by 10.9 mm^2^) relative to that on the wounding day (*p* < 0.05). That is, in the Control_0 group, the acute inflammatory period lasted ≤3 days, and the decrease in the wound size began from day 5. By day 7, the average intact wound area was 23.9% less than the initial area (*p* < 0.01).

In the Control_NaCl group, the injection of 0.9% NaCl solution caused additional injuries. The period during which wound area expansion occurred increased by ≥2 days relative to the Control_0 group. The wound area of the Control_NaCl group increased by 27.0% on day 1, which was 9.3% more than the increase in the Control_0 group. On day 3, the wound area of the Control_NaCl wounds was 25.6% more than the initial area and 26% more than the Control_0 group. These differences were statistically significant (*p* = 0.001). On day 5, the wound area of the Control_NaCl group was 13.6% greater than the initial area, which was 23% greater than in the Control_0 group on average on the same day (*p* = 0.007). On day 7, the wound area of the Control_NaCl group had returned to its initial size, decreasing by only 1.9% (2.2 mm^2^) on average relative to that on the wounding day. Regeneration was 22% slower than that in the Control_0 group (*p* = 0.034).

**Figure 3:**
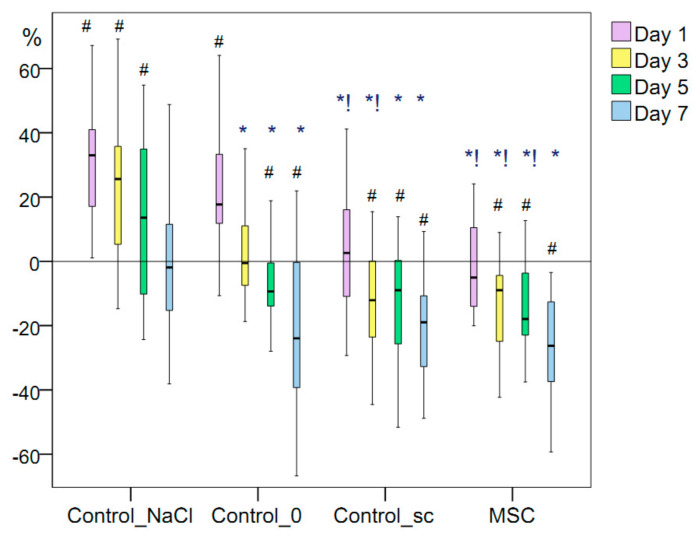
Changes in wound area (%). Note that for the data presented: #, difference in the dynamics of the associated indicator within a particular group relative to day 0 at *p* < 0.05 (Wilcoxon test); Kruskal–Wallis test: *p* = 0.0001 on day 1, day 3, day 5 and day 7; *, difference from the Control_TфCд group at *p* < 0.05; !, the difference from the Control_TфCд group at *p* < 0.05.

The additional wound trauma caused by the injection of 0.9% sodium chloride solution into the wound edges on the day of modeling mimicked the local infiltration of anesthesia into wounds that occurs during primary surgical treatment and small surgical operations, and results in a prolonged period of wound-tissue expansion during the inflammation period. The wound area maintained its maximum size ≤5 days at a statistically significant level relative to that on day 0 of the experiment and returned to its original size by day 7. Tight infiltration caused additional damage to the microvasculature and the direct intercellular connections. This damage slowed the regeneration process and resulted in an increase in the wound area in the Control_NaCl group by 22–26% relative to that in the Control_0 group (*p* < 0.05) on days 1, 3, 5, and 7 (Figure 3).

We emphasize that the simultaneous comparison of all four groups using the Kruskal–Wallis test revealed a statistically significant difference in the wound area change at all time points of the study (*p* = 0.00001). This allows us to discuss the different mechanisms of wound healing.

MSCs injection into the wound edges on the day of their modeling demonstrated its effectiveness from the first day of the experiment and throughout the entire inflammation period despite the trauma it also caused. Twenty-four hours after modeling, the size of the wounds in the MSC group decreased by 5% on average. Thus, more than half of the rats showed decreases in the wound-tissue area and an absence of an inflammatory expansion period, which was not observed in the Control_0 or Control_NaCl groups on day 1. However, the wounds of the Control_sc group, which were located on the opposite side of the MSC group rats, showed areas that increased by 2.6% on average on day 1. We hypothesized that the decrease in the area of wounds in the MSC group compared to the control in the first three days may be due to a decrease in edema, which can be explained by the anti-inflammatory effect of MSCs. This means that more than half of the rats showed expansion in the wound area within 24 h. The difference in wound areas between the MSC and Control_sc groups within 24 h was statistically significant (*p* = 0.045). We recorded a significant improvement in wound healing in the Control_sc group relative to that in the Control_NaCl and Control_0 groups within the first day (*p* < 0.05), which supports the hypothesis that treatment with MSCs should have a positive general effect without additional injury to wounds.

**Figure 4:**
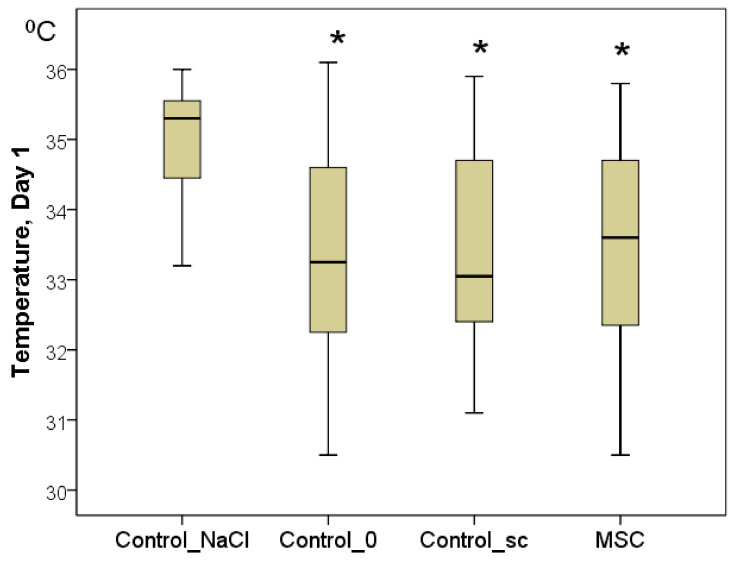
The temperature of the wound centers in different groups on day 1, in degrees Celsius (Kruskal–Wallis test: *p* = 0.0001; * difference from the Control_NaCl group at *p* < 0.05).

To confirm the latter point, the temperature of the center of the wounds was measured. Although the background values were indistinguishable between the groups on day 1, statistically significant differences were already established by day 1. In every group, the wound temperature increased significantly relative to day 0 (*p* < 0.05). Nevertheless, the highest increase in the wound center temperature was recorded in the Control_NaCl group (from 32.0 to 35.3 °C on average; *p* < 0.05). Wound center temperatures were 33.3 °C in the Control_0 group (2.0 °C less on average than in the Control_NaCl group; *p* < 0.05), 33.1 °C in the Control_sc group (2.2 °C less on average than in the Control_NaCl group; *p* < 0.05), and 33.6 °C in the MSC group (1.7 °C less on average than in the Control_NaCl group; *p* < 0.05). We found no significant differences among the latter three groups on day 1 (Figure 4). Subsequently, statistical differences among the groups were not found for this indicator (*p* > 0.05).

The wound areas were not significantly different on average between the Control_sc and MSC groups on day 3, decreasing by 12% and 9%, respectively. The result was significantly better in the MSC group than in the Control_0 group (*p* < 0.01) and Control_NaCl group (*p* < 0.01). Therefore, the effect of MSCs (either remote or local) was positive, accelerating regeneration of the same-size wounds by day 3.

On day 5 of the study, the wound area of the Control_sc group decreased by 9.0% on average relative to the initial size of the wound on the wounding day, which was comparable with the decrease in the Control_0 group (*p* > 0.05). The area reduction was 17.9% on average in the MSC group, which was 1.90-fold higher than that in the Control_0 group (*p* < 0.05) and 31.5% higher than that in the Control_NaCl group (*p* < 0.01).

On day 7 of the study, the wound area of the Control_sc group decreased by 18.9% on average relative to its initial size, and the wound area of the MSC group decreased by 26.3%. This was significantly different from the decrease in the Control_NaCl group by 9.1- and 13.8-fold on average (*p* < 0.01 for both), respectively, but was not significantly different from the Control_0 group (*p* > 0.05).

Therefore, the anti-inflammatory effect of the local injection of stem cells into the wound was observed for 24 h, after which the decrease in its external effects was offset by an increase in microhemocirculation. During days 3–7, we found no statistically significant differences in the wound areas between the MSC and Control_sc groups. The intact wounds of the Control_0 and Control_sc groups statistically differed only during days 1–3, whereas the wounds in the MSC group and the wounds with additional damage (Control_NaCl group) differed statistically in area during all acute inflammatory periods. In addition, on the first day, the wound of the MSC group was the same size as on day 0. From day 3 onward, the wound area decreased significantly, with the best results achieved on days 5 and 7 with local injection of MSCs.

### 3.2. Weight Changes

The first series of rats (Control_0 and Control_NaCl groups) weighed ≥7 g more on average (*p* > 0.05) 24 h after modeling; they had lost 25 g on day 3 and weighed significantly less on day 5 than on the wounding day (49 g on average, *p* < 0.05). On day 7, the weight of the control rats returned to near their initial levels as they had only lost an average of 1 g (*p* > 0.05).

The weight of the rats treated with MSCs did not significantly change throughout the experiment (changes in wound area, *p* > 0.05); on day 7, the rats weighed 14 g more on average than on the wounding day (*p* > 0.05). On day 5, a slight but non-significant decrease in weight was recorded (loss of 5 g on average relative to day 0, *p* > 0.05). In the second series of rats, those treated with MSCs showed a significant weight increase of 16 g (*p* < 0.05) on day 5.

**Figure 5:**
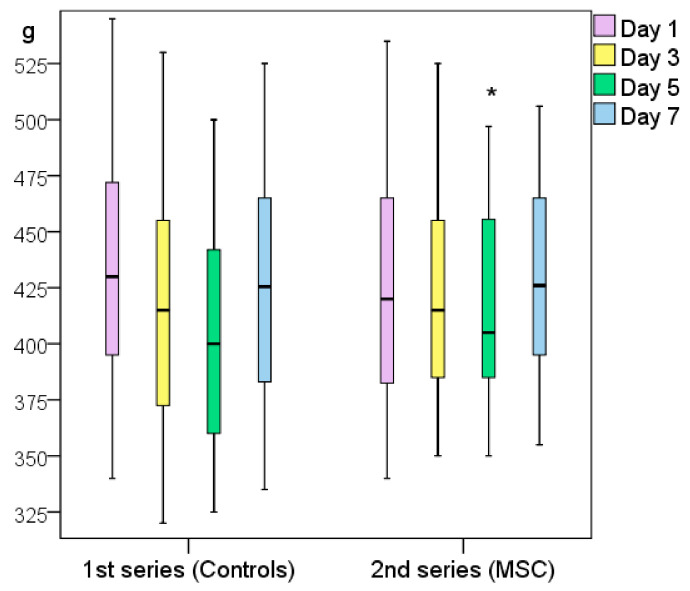
Weight changes of the two experimental series of rats (first series, Control_0 and Control_NaCl groups; second series, MSC and Control_sc groups). Note: * difference between groups at *p* < 0.05 (Mann–Whitney U-test).

To summarize, on day 5, the weight of the control animals decreased significantly, but by day 7, they returned to their day-0 weight. This may indirectly indicate the end of the acute inflammatory period and beginning of the regeneration process. The weight of the animals in the MSC group did not show any significant variations during the acute inflammation phase, which can be attributed to the advantages of using MSCs. Notably, the rats in the second series weighed less than the rats in the first series at the beginning of the experiment (difference not statistically significant), but the trend was clear. Still, by day 5, the second series of rats weighed more than the first series because their more active metabolic processes increased the rate of wound healing (Figure 5).

### 3.3. Blood Perfusion Dynamics in the Wound-Edge Skin

The microvessel perfusion volume in the wound-edge skin during the first minutes after wounding was the same in all four groups on average (median): 122 BPU in the Control_NaCl group, 159 BPU in the Control_0 group, 137 BPU in the Control_sc group, and 118 BPU in the MSC group (*p* > 0.05 for all groups). By day 3, the volume of microcirculation remained unchanged and decreased in the Control_NaCl (median = 115 BPU), Control_0 (median = 134 BPU), and Control_sc (median = 127 BPU) rats. The only increase, which was insignificant, was the median of this indicator in the MSC group (median = 121 BPU). We found no significant differences in blood perfusion volumes between the groups (*p* > 0.05) on day 3.

**Figure 6:**
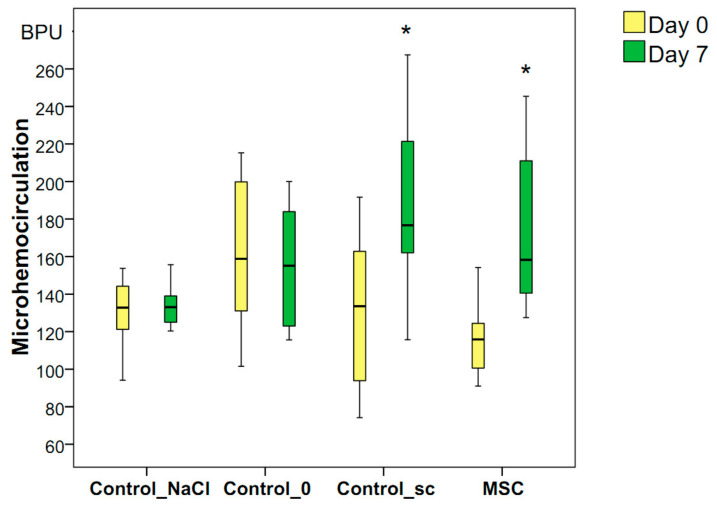
Microhemocirculation in the wound-edge tissues of different groups on days 0 and 7 in different groups; * difference in the dynamics of the associated indicator in the group relative to day 0 at *p* < 0.05 (Wilcoxon test).

On day 7, an increase in the blood flow volume in the microhemocirculation vessels of the wound tissues was recorded relative to the background level of the MSC group (the indicator increased 1.34-fold, ≤158 BPU, *p* < 0.05) and Control_sc group (the indicator increased 1.28-fold, ≤176 BPU, *p* < 0.05). In other groups, the blood flow volume remained unchanged on day 7 compared to day 0, and averaged 133 BPU in the Control_NaCl group and 145 BPU in the Control_0 group (*p* > 0.05). Thus, we established the local and general effects of MSCs on the increase in the microcirculation levels on day 7 after wounding, despite the lack of significant differences in the hemoperfusion index on day 7 between the groups studied (*p* = 0.316 according to the Kruskal–Wallis test; Figure 6).

### 3.4. Histological Analysis Results

Day 3 marked the beginning of the exudative inflammation phase, during which increases in leukocyte infiltration and tissue edema were recorded. The most acute inflammation was observed in the Control_sc group; the least was in the MSC group. The number of leukocyte cells was the lowest in the SC group wound centers (median = 179 cells/mm^2^): on average, in the Control_NaCl group, this indicator was 1.17-fold higher on day 3; in the Control_0 group, it was 1.10-fold higher; in the Control_sc group, it was 1.28-fold higher (all *p* < 0.05). The same trend was observed for the wound edges (median = 130 cells/mm^2^): in the Control_NaCl group, this indicator was on average 1.35-fold higher on day 3; in the Control_0 group, it was 1.22-fold higher; in the Control_sc group, it was 1.35-fold higher (*p* < 0.05). The leukocyte numbers were higher than the fibroblast numbers on day 3 in all wound areas. The fibroblast-to-leukocyte ratio was the same in the groups studied and averaged 0.55–0.59 (*p* > 0.05) at the bottom of the wound. However, at the wound edges, the ratio was significantly different. The fibroblast-to-leukocyte ratio was highest in the Control_NaCl group (median = 0.60), which was 1.46-fold higher than in the Control_sc group (*p* < 0.05), 2.14-fold higher than in the Control_0 group (*p* < 0.05), and 3.16-fold higher than in the MSC group (*p* < 0.05). The difference in the ratios between the MSC and Control_sc groups on day 3 was 2.16-fold (*p* < 0.05), the same as for the wounds in the first series of experiments (Table 1). Probably, additional trauma by injection in the Control_NaCl group was caused by inflammation; however, the effect of inflammation as a result of additional trauma after injection of MSCs stabilized, despite the similar volume and method of injection trauma. We explain this by the therapeutic effect of biologically active substances synthesized by MSCs, since the effect of inhibiting inflammation lasted several days.

In each case, tissue regeneration proceeded faster at the wound edges than at the bottom of the wounds due to the presence of a prepared functioning cell mass and an existing microcapillary bed. Bottom tissue regeneration always lags relative to external wound regeneration because of the time required for creation of the extracellular matrix and the local capillary system.

**Figure 7:**
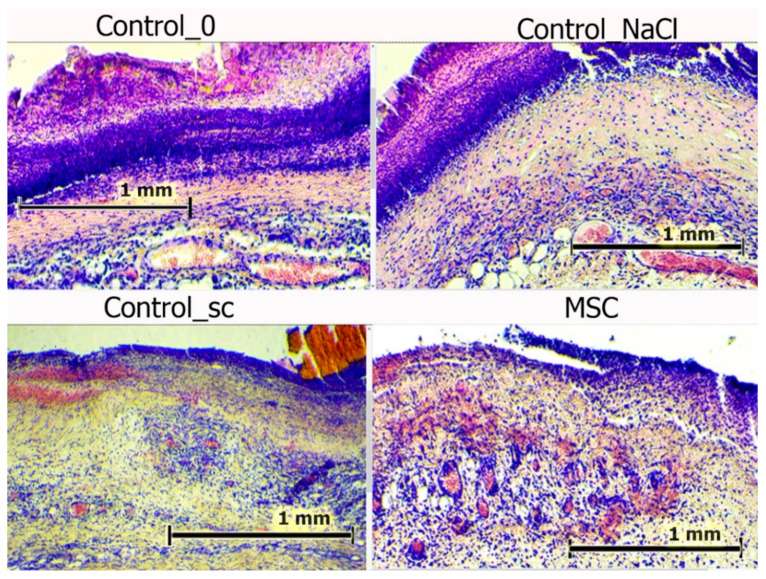
Wounds centers in different groups on day 3. Hematoxylin and eosin staining (100×). The scale bar is 1 mm.

All the wounds were covered with scabs on day 3. The scabs were broader in the control groups, with a maximum area observed in the Control_NaCl group. Deep sections of the scab were infiltrated with polymorphic nuclear leukocytes. However, this infiltration was fundamentally different. In the wounds of the Control_NaCl group, there was a stable, pronounced leukocyte infiltration. This was not typical of the day of intact wounds in the Control_0 and Control_sc groups. The infiltrates spread into the granulation tissue and deeper dermis layers. Whereas the scab areas were smallest and their impregnation with leukocytes the least in the MSC group, the granulation and the underlying layers of tissue edema were high. Blood stasis was observed in all wounds. Vascular congestion of the microvasculature was less explicit in the intact wounds of the Control_0 group and particularly those of the Control_sc group (Figure 7).

Thus, on day 3, we assumed that MSCs injected into the wound edges produced some effect that inhibited phagocytic function and reduced their migration to the wound edges. This migration was even less than in the Control_0 group.

**Figure 8:**
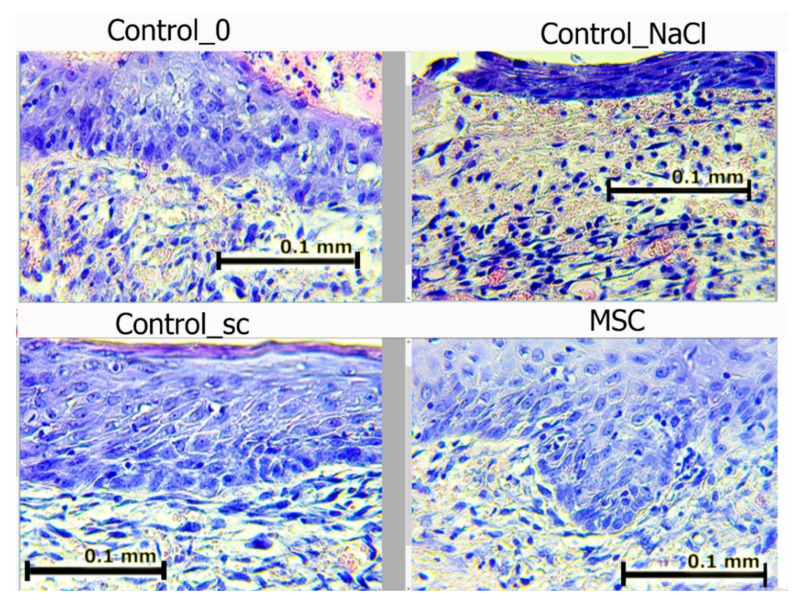
Wound edges in different groups on day 7. Hematoxylin and eosin staining (400×). The scale bar is 0.1 mm.

On day 7, we noted epidermis regeneration from the edges to the wound center in all groups. The most organized marginal shaft of the epidermis was observed in the SC and Control_sc groups (full layer of epidermis with a basement membrane, under which the papillary dermis formed). The epidermis was most completely formed in the MSC group, where the growth buds from which the skin-tissue derives (including hair follicles) would soon form were often identified. In wounds in the Control_NaCl and Control_0 groups, the exudation-phase continuation phenomenon was observed. The number of leukocytes in the wound edges and center was the highest among these groups. In all of the wounds, the leukocyte infiltration density and fibroblast maturity gradients moved from the inside to the outside (Figure 8).

Figure 8 shows that in groups Control_0 and Control_NaCl, leukocyte cells with a round shape prevailed; in groups with MSCs, fibroblasts predominated—cells of irregular, spindle-shaped (fibrocytes) or stellate cells with processes that produced collagen (active fibroblasts).

The total number of fibroblasts and leukocytes increased significantly by day 7 in all groups (Table 2).

At the wound edges, the number of cells increased 2-fold on average (the greatest increase, 2.6-fold, was observed in the SC group); in the wound centers, the number of cells increased 4-fold on average (the greatest increase, 5.6-fold, was observed in the SC group, while the least increase, 3.0-fold, was observed in the Control_0 group; *p* < 0.05 for both groups). The number of fibroblasts increased to a greater extent, with the largest number recorded in the wound centers of the SC group (median = 449 fibroblasts/mm^2^). However, we observed significant differences in the number of fibroblasts found in the wound edges. Additionally, an important indicator was the ratio of fibroblasts-to-leukocytes. In the wound centers, despite the absence of any significant differences among the groups, the ratio was highest because the number of fibroblasts was largest and the number of leukocytes was smallest in the SC group (median = 1.57) compared to the other groups. In contrast, at the wound edges, the fibroblast-to-leukocyte ratio in the Control_NaCl group was significantly lower than those in the other groups (on average, 2.1- to 2.3-fold; *p* < 0.05); the greatest number of leukocytes and least number of fibroblasts in the wound edges were observed because of the continuation of the exudative phase and a delay in the transition to the proliferation phase, respectively.

## 4. Discussion

The continuing debate about the efficacy of using MSCs in the treatment of human diseases began with the development of technology that allows the cell culture of these cells and the direct differentiation of the cultures. To date, numerous studies and systematic reviews have attempted to evaluate the effect of MSCs on various somatic diseases, yet more questions than answers remain. One of the most effective methods is to study MSCs in animals; however, this method has one significant drawback: from a practical standpoint, no experimental models exist that reflect the real etiopathogenetic roots of diseases. The simplest wound models are the most informative regarding the use of MSC, methods of cell introduction and their quantity, and use of auto- or allocultures [23,24,25,26,27,28,29,30,34].

In the course of our research in elderly rats, we identified that the inflammatory period in the untreated wounds lasted significantly longer except when using MSC, and that positive changes were noted only starting from day 5. Infiltration with isotonic sodium chloride solution further slowed the processes of skin wound recovery and prolonged the inflammatory period up to 5–7 days. These differences may be explained by the tight infiltration of the wound edges leading to additional damage to the direct intercellular connections and to the microvasculature. This raises the question of the feasibility of injecting drugs and of infiltrative anesthesia during wound treatment without appropriate prescriptions and an objective risk–benefit assessment.

Treatment with MSCs doubled these indicators, demonstrating an effect following application, even on the first day of treatment. The strength and duration of a single local effect of MSC were greater than its general effect. However, the differences in the size of wounds between the groups that received general versus local application of MSCs were not apparent until 24 h after treatment; later, the changes in the area of the wounds did not differ between the groups.

It is also important to consider the paracrine effect, but in the context of its therapeutic effect and the possibility of using MSCs in the clinic, the paracrine effect does not matter. This means that, in some cases, the recommendation would be to use remote (not local) introduction of MSCs. For example, during plastic and cosmetic surgery, when additional damage to the skin is inappropriate, a possible solution would be to use general MSCs application. Notably, the efficacious period of the remote effect in elderly animals was limited to 3–5 days, so repeated general administration of MSC every 5 days is recommended to achieve the best regenerative effect. This assumption requires further verification. Nevertheless, the results obtained convincingly showed that the effect exerted even by the allogeneic cells of another biological species is the same as when using autologous cells in other published works [23,24,25,26,27]. In addition, the remote effect of accelerating regeneration is obviously universal, regardless of the age of the animals. It can be assumed that the degree of regeneration stimulation depends on the rate of the body’s metabolic processes, but this requires additional consideration.

The undeniable feasibility of local administration of MSCs implies its periodicity. During the first 5 days, the wound healing dynamics showed better progress in the MSC group than in the Control_0 group, after which they stabilized. Therefore, we assume that the additional injection of MSCs into a wound every 5–10 days would allow wound healing to occur faster than wound regeneration.

The significant increase in the microhemocirculation volume of the wound-edge skin on day 7, which was recorded only in the wounds of the MSC and Control_sc groups, allows us to conclude that both the locally administered and general effects of MSC were identical in strength and contributed to accelerated wound healing.

By the end of day 7, the mechanisms of granulation tissue formation stimulated advanced acceleration in wound-tissue regeneration. Development of hair follicles and sebaceous glands were observed in the MSC group. Another indication of the increase in overall metabolism was the stability of the weight of the animals in the MSC series compared to that in the control rats, which weighed less.

When summarizing the results of our experiments, it can be argued that in the model of acute skin injury, MSCs showed that they share the common mammalian mechanisms that stimulate wound regeneration. This was confirmed by a shortening of the inflammation period and an early transition to the restoration of full-fledged skin due to an increase in the concentration of fibroblasts synthesizing the intercellular matrix and maximum preservation of the microcapillary channels in the wounds. The strengthening of general metabolic processes was illustrated by an increase in the body weight of the animals in the MSC group and the concomitant fastest wound closure. Metabolic growth is provided not by the cells themselves, but by the biologically active substances they synthesize; in the Control_sc group, it already began within the first day and cannot be explained only by homing. The actions of the introduced allogeneic MSCs lasted no more than a week and ended with cell death, apparently due to the immune response of the host organism in response to the onset of their differentiation and the appearance of the effect of histone incompatibility.

## 5. Conclusions

Treatment with MSCs contributed to the inhibition of pathophysiological inflammatory processes in the acute phase and improved wound regeneration on days 1–3 in the remote group and days 1–5 in the local (injection) group. On the seventh day, all the studied parameters of the wounds of the Control_0 group were the same in those of the wounds that received cell therapy. The results obtained may help explain why the effectiveness observed in tissue culture research has not yet provided the basis for a practical medical breakthrough.

Since the maximum efficacy of MSCs is limited to the first 5 days, we recommend investigating the efficacy of MSC applied every 5–10 days after injury to discover whether this significantly enhances the isolated effect observed after a single injection of MSCs. As additional injection trauma to wounds slowed the regeneration process, and considering the comparability of the local versus the general action of MSCs in the dynamics of wound size, we think that MSCs should be used without any additional injury to the wounds. The remote effect of treatment with MSCs is especially useful in the treatment of multiple wounds.

## 6. Features of the Approach of the Study

We investigated the efficacy of using progenetic MSCs cultured from human umbilical cords in wound healing in rats, introduced via local (direct injection into the wound) or remote application to intact wounds in rats. Using 56-day-old rats, a multifactorial randomized experiment was performed with the analysis of histological, morphometric, and microcirculatory parameters of lamellar cutaneous acute wounds, with an assessment of the components of exudative inflammation and transition to proliferation, in four study groups (the results of the study of local and gene-based cell therapy were simultaneously intact and invasive control). The morphometric composition of fibroblast and leukocyte cells in different parts of the wounds (center and edges) was studied separately; the decisive involvement of leukocyte infiltration and fibroblast proliferation in the development of different stages of inflammation was demonstrated, and regularities that accelerate the processes of regeneration were revealed.

## 7. Research Limitations

We understand that our work is not devoid of some shortcomings. For example, it would be interesting to evaluate the dynamics of the level of growth factors in the blood and tissues of the animals on a daily basis by marking the stem cells and assessing their migration, including to the second (opposite) wound. We did not aim, in this study, to examine the immunological parameters in standardized Wistar rats under standard vivarium conditions, although in the case of bacterial contamination and experimental sepsis, this would be interesting to determine. It would also be interesting to continue the study until day 90–180 and evaluate the course and outcome of remodeling, including scar formation. However, no research, especially in biology and medicine, can be final and ideally aligned due to the considerable complexity of the systems under study. Therefore, we think that not answering some of the questions in any of the studies only increases the number of new questions. The new questions raised in the course of our research will be the basis for our subsequent work.

## Figures and Tables

**Table 1:** Number of fibroblasts and leukocytes in the wound edges of the different groups on day 3 (n).

	Control_0 (0)	Control_NaCl (1)	Control_sc (C_sc)	MSC	Kruskal–Wallis Test	*p* < 0.05 (Mann–Whitney Test)
Fibroblasts numbers in the wound center	119 113/120	117 108/121	133 101/142	98 92/117	*p* = 0.193	MSC/C_sc
Leukocytes numbers in the wound center	197 189/241	210 207/217	229 187/263	179 162/184	*p* = 0.045	0/MSC 1/MSC MSC/C_sc
Fibroblasts to leukocytes ratio in the wound center	0.59 0.49/0.61	0.57 0.50/0.59	0.56 0.49/0.66	0.55 0.52/0.66	*p* = 0.700	---
Fibroblasts numbers in the wound edges	47 43/54	107 92/110	72 47/78	18 14/49	*p* = 0.002	0/1 0/C_sc 1/C_sc 1/MSC MSC/C_sc
Leukocytes numbers in the wound edge	159 145/229	175 164/181	176 151/188	130 102/139	*p* = 0.041	0/MSC 1/MSC MSC/C_sc
Fibroblasts to leukocytes ratio in the wound edge	0.28 0.24/0.29	0.60 0.57/0.61	0.41 0.32/0.44	0.19 0.12/0.36	*p* = 0.001	0/1 0/C_sc 1/C_sc 1/MSC MSC/C_sc

Note: the 1st line is the median; the 2nd line is the lower and upper quartiles.

**Table 2:** Number of fibroblasts and leukocytes in the wound center and wound edges of different groups on day 7 (n).

	Control_0 (0)	Control_ NaCl (1)	Control_sc (C_sc)	MSC	Kruskal–Wallis Test	*p* < 0.05 (Mann–Whitney Test)
Fibroblasts numbers in the wound center	420 375/445	428 383/434	422 411/462	449 395/458	*p* = 0.787	MSC/C_sc
Leukocytes numbers in the wound center	327 256/378	303 276/309	298 261/333	279 255/292	*p* = 0.479	---
Fibroblasts to leukocytes ratio in the wound center	1.23 1.13/1.47	1.46 1.39/1.56	1.40 1.33/1.66	1.57 1.54/1.64	*p* = 0.330	---
Fibroblasts numbers in the wound edges	682 668/691	538 448/549	608 570/643	594 574/638	*p* = 0.001	0/1 0/C_sc 0/MSC 1/C_sc 1/MSC
Leukocytes numbers in the wound edge	204 185/248	349 325/383	180 171/218	186 176/219	*p* = 0.001	0/1 1/C_sc 1/MSC
Fibroblasts to leukocytes ratio in the wound edge	3.34 2.65/3.69	1.47 1.32/1.60	3.38 2.63/3.76	3.14 2.84/3.38	*p* = 0.001	0/1 1/C_sc 1/MSC

Note: the 1st line is the median; the 2nd line is the lower and upper quartiles.

## Data Availability

Data confirming the results obtained can be found by contacting the author (silinaekaterina@mail.ru).
